# Influence of Annealing on the Properties of Fe_62_Ni_18_P_13_C_7_ Alloy

**DOI:** 10.3390/ma18143376

**Published:** 2025-07-18

**Authors:** Aleksandra Małachowska, Łukasz Szczepański, Andrzej Żak, Anna Kuś, Łukasz Żrodowski, Łukasz Maj, Wirginia Pilarczyk

**Affiliations:** 1 Faculty of Mechanical Engineering, Wroclaw University of Science and Technology, Wybrzeże Wyspiańskiego 27, 50-370 Wroclaw, Poland; 2 Institute of Advanced Materials, Faculty of Chemistry, Wroclaw University of Science and Technology, Wybrzeże Wyspiańskiego 27, 50-370 Wroclaw, Poland; andrzej.zak@pwr.edu.pl; 3 AMAZEMET Sp. z o. o. [Ltd.], Al. Jana Pawła II 27, 00-867 Warsaw, Poland; lukasz.zrodowski@amazemet.com; 4 Faculty of Materials Science and Engineering, Warsaw University of Technology, ul. Wołoska 141, 02-507 Warsaw, Poland; 5 Institute of Metallurgy and Materials Science, Polish Academy of Sciences, ul. Reymonta 25, 30-059 Krakow, Poland; l.maj@imim.pl; 6 Faculty of Mechanical Engineering, Silesian University of Technology, ul. Akademicka 2A, 44-100 Gliwice, Poland; wirginia.pilarczyk@polsl.pl

**Keywords:** metallic glasses, crystallization, ribbons, annealing, devitrification

## Abstract

In this study, the influence of annealing on the phase evolution and mechanical properties of the Fe_62_Ni_18_P_13_C_7_ (at.%) alloy was investigated. Ribbons produced via melt-spinning were annealed at various temperatures, and their structural transformations and hardness were evaluated. The alloy exhibited a narrow supercooled liquid region (ΔT_x_ ≈ 22 °C), confirming its low glass-forming ability (GFA). Primary crystallization began at approximately 380 °C with the formation of α-(Fe,Ni) and Fe_2_NiP, followed by the emergence of γ-(Fe,Ni) phase at higher temperatures. A significant increase in hardness was observed after annealing up to 415 °C, primarily due to nanocrystallization and phosphide precipitation. Further heating resulted in a hardness plateau, followed by a noticeable decline. Additionally, samples were produced via selective laser melting (SLM). The microstructure of the SLM-processed material revealed extensive cracking and the coexistence of phosphorus-rich regions corresponding to Fe_2_NiP and iron-rich regions associated with γ-(Fe,Ni).

## 1. Introduction

Iron-based metallic glasses are extensively studied materials due to their high mechanical strength, good corrosion resistance, excellent soft magnetic properties, and relatively low production cost [1]. Traditionally, they are produced in the form of ribbons. This is related to the simplicity of the fabrication process and the low requirements for glass forming ability (GFA) [1]. Pauly et al. [2] demonstrated that SLM can produce components from an Fe-based metallic glass, taking advantage of the high cooling rates inherent in the process. In practice, however, the production of high-quality samples from iron-based metallic glasses remains a significant challenge [3,4,5]. The most widely researched problems are cracks, pores, and crystallized regions [6]. The crystallization tendency of metallic glasses with low GFA can be mitigated through the application of optimized scanning strategies [7], while crack formation may be reduced by improving the plasticity of the material [8]. Unfortunately, most Fe-based metallic glasses exhibit negligible plastic deformation at room temperature, which, in combination with the high thermal gradients generated during laser processing, promotes crack initiation and propagation. To address this limitation, Zou et al. [9] proposed the incorporation of ductile copper particles into a FeCrMoCB metallic glass matrix, which effectively suppressed crack formation during SLM processing. Another promising approach involves the in situ formation of ductile phases. For example, Fe_77_Mo_5_P_9_C_7.5_B_1.5_ bulk metallic glass (BMG) reinforced with in situ formed ductile α-Fe dendrites exhibited a plastic strain exceeding 30% and a fracture strength above 3.0 GPa [10]. This strategy has also been successfully demonstrated for SLM-processed Zr/Ti-based metallic glasses [11].

One of the popular Fe-based metallic glass systems is the Fe–P–C, which was later studied for partial substitution of Fe with Ni, primarily with the objective of improving the compressive plasticity of bulk metallic glasses (BMGs) [12]. Fe-Ni-P-C systems have been reported to have exceptional plasticity. For instance, Guo et al. [13] reported an engineering plastic strain exceeding 50% for Fe_62_Ni_18_P_13_C_7_ alloy under quasi-static uniaxial compression. Similarly, Sarac et al. [14] reported up to 50% plastic strain for a similar Fe_50_Ni_30_P_13_C_7_ composition under uniaxial compression (unfortunately, no tensile data were available due to sample geometry limitations). The enhanced plasticity was attributed to the presence of nanometer-sized γ-FeNi crystals homogeneously distributed within the soft matrix. It has also been shown that increasing the Ni content in Fe-Ni-P-C systems reduces GFA and promotes nanocrystal formation, which leads to local phosphorus enrichment and its subsequent ejection in the form of clusters [14]. Consequently, the addition of phosphorus decreases the embrittlement temperature [15]. Although the Fe-Ni-P-C systems have relatively low GFA (the reported critical diameters reached a maximum of 2.5 mm [16]), it has been shown that the crystallization tendency of metallic glasses with low GFA can be mitigated through the application of optimized scanning strategies [7]. Additionally, the uniformly nanocrystallized alloy may still possess good magnetic properties [17].

This study investigates the thermal stability and annealing behavior of melt-spun Fe_62_Ni_18_P_13_C_7_ (at.%) ribbons. While Fe-based bulk metallic glasses and Fe–Ni–P–C systems have been previously investigated [12,14], a systematic evaluation of their microstructure and properties—combined with a feasibility study of selective laser melting (SLM)—has not yet been reported. The ribbons were annealed at various temperatures, and phase evolution, microstructure, and hardness were characterized. The objective was to determine the influence of annealing on mechanical properties, particularly hardness, and to clarify the crystallization mechanisms involved. For comparison, preliminary experiments involving selective laser melting (SLM) of the same alloy were also conducted. The results from SLM processing are presented as supplementary observations, while the primary focus remains on the outcomes of the annealing experiments.

## 2. Materials and Methods

The base alloy ingot, with a nominal composition of Fe_62_Ni_18_P_13_C_7_ (at.%), was prepared by induction melting. The atomic percentages were first converted to weight percentages. Based on these values, high-purity elemental Fe, Ni, P, and C (≥99.9%) (Onyxmet Tomasz Olszewski, Olsztyn, Poland) were precisely weighed and then melted together in an induction furnace under an argon atmosphere to ensure homogeneity of the alloy. Subsequently, the ribbons were produced using the melt-spinning technique (Melt Spinner SC, Edmund Bühler GmbH, Bodelshausen, Germany), which enables ultrafast cooling of the molten alloy under vacuum conditions [18]. The melt-spinning process was carried out under the following conditions: the roller rotation speed was set to 20 m/s, and the casting was performed at a temperature of 1100 °C. The system was initially evacuated to a vacuum level of 7 × 10^−2^ mBar and then pressurized to a working pressure of 800 mBar. The injection pressure was 400 mBar, with the distance between the crucible and the roller maintained at 0.3 mm. The crucible used had a diameter of 20 mm.

The thickness of the cast ribbons was at the level of 30 μm. The cast ribbons were cut into seven samples, each 100 mm in length. Six samples were annealed at 310, 380, 415, 470, 510, and 570 °C for 10 min in an Lt15/12/C450 furnace (Nabertherm GmbH, Lilienthal, Germany). The annealing is a standard method to study phase evolution [19]. The annealing temperatures in this study were selected based on the characteristic transformation ranges identified by DSC analysis, as well as the temperatures at which microstructural changes were observed during the in situ TEM heating experiments described below. One sample was retained in the as-cast state and served as a reference. Additionally, the Selective Laser Melting (SLM) process was carried out using gas-atomized Fe_62_Ni_18_P_13_C_7_ powder with a particle size distribution of +20/−63 µm, supplied by NANOVAL GmbH & Co. KG (NANOVAL GmbH & Co. KG, Berlin, Germany). The printing process was carried out on an AISI 316 substrate using a Realizer SLM 50 system (Realizer GmbH, Borchen, Germany) equipped with a 120 W continuous-wave 1064 nm fiber laser. The parameters were selected based on the experience with low GFA metallic glass alloy [7]; as reported, the application of a “random hatch scanning” strategy prevents local overheating and ensures uniform heat distribution throughout the sample.

The fabrication procedure consisted of two stages [20]. In the first stage, referred to as “premelting”, an XY scanning strategy was employed with a laser speed of 125 mm/s, laser power of 30 W, and a hatch distance of 120 µm. Successive layers were rotated by 90 degrees to ensure uniform bonding. The second stage, “remelting”, was performed at a scanning speed of 3000 mm/s, laser power of 120 W, and a hatch distance of 30 µm. This step was essential for achieving a high amorphous phase fraction and could only be effectively implemented following the premelting stage, as spatter generation would otherwise prevent successful processing. The samples were fabricated as chamfered blocks with dimensions of 15 × 15 × 4 mm, directly attached to the build plate to minimize the risk of print delamination and to enhance heat dissipation. The build plate was made of AISI 304 stainless steel.

The phase composition of the samples was determined using X-ray diffraction (XRD) with a Bruker D8 Advance diffractometer (Bruker AXS, Karlsruhe, Germany) employing Cu-Kα radiation (λ = 1.5406 Å). Data were collected over a 2θ range of 20° to 80° with a step size of 0.02°.

Hardness measurements were performed via nanoindentation using an NHT3 nanoindenter (Anton Paar TriTec SA, Corcelles, Switzerland) equipped with a Berkovich diamond tip. The tests were conducted under a maximum load of 50 mN and a loading rate of 100 mN/min. Hardness values were calculated according to the Oliver–Pharr method. For each sample, ten measurements were performed, and the reported value represents the arithmetic mean.

Differential scanning calorimetry (DSC) analysis was conducted using a DSC 8500 instrument (PerkinElmer Inc., Waltham, MA, USA) with a heating rate of 10 °C/min, up to a maximum temperature of 600 °C, under a nitrogen atmosphere. The relative amorphous phase content (*P*) in the heat-treated ribbons was determined by comparing the exothermic enthalpy of heat-treated ribbons (*ΔH_heat_treated_ribbon_*) to that of the as-cast ribbon (*ΔH_as_cast_ribbon_*) and it was calculated using the formula *P* = *ΔH_heat_treated_ribbon_*/*ΔH_as_cast_ribbon_* × 100 *pct* [21].

Samples for metallographic analysis were sectioned from the printed specimens and prepared using standard metallographic procedures. The microstructure of the printed samples was examined using a Keyence VHX-6000 (Keyence Corporation, Osaka, Japan) digital microscope.

The samples for transmission electron microscopy (TEM) analysis were initially punched into 3 mm disks using a Disc Punch (Gatan Inc., Pleasanton, CA, USA). Since the as-cast ribbons had a thickness of approximately 30 µm, no mechanical pre-thinning was required. Final thinning was performed by electrolytic polishing with a TenuPol (Struers A/S, Ballerup, Denmark), followed by a short ion polishing step (2 min) using a DuoMill system (Gatan Inc., Pleasanton, CA, USA) to remove surface contamination and residual polishing artifacts. TEM observations of the ribbons were carried out using a Hitachi H-800 (Hitachi High-Tech Corporation, Tokyo, Japan) transmission electron microscope equipped with a custom-designed Hitachi heating holder. For in situ heating experiments, the samples were heated from room temperature to 570 °C at a rate of approximately 10 °C/min. Selected Area Electron Diffraction (SAED) patterns were analyzed using the open-source software CrysTBox v.1.1 [22].

For nanoscale microstructure investigations, a ThermoFisher Themis G2 200 kV (Thermo Fisher Scientific, Waltham, MA, USA) analytical electron microscope (STEM) was used. This microscope was equipped with a Ceta camera (Thermo Fisher Scientific, Waltham, MA, USA) for bright-field imaging, a Fischione high-angle annular dark-field STEM detector, and an energy-dispersive spectroscope (EDS) using a ChemiSTEM (Thermo Fisher Scientific, Waltham, MA, USA) for chemical composition analysis. The samples were prepared using the focused ion beam (FIB) technique, utilizing a ThermoFisher Scios 2 Dual Beam (Thermo Fisher Scientific, Waltham, MA, USA) microscope with a Ga+ ion source and an EasyLift lift-out system. The FIB-prepared lamellae were mounted on a copper grid using a Pt gas injection system and thinned to approximately 100 nm to ensure electron beam transparency in the TEM.

## 3. Results

### 3.1. Phase Transformation

**Figure 1:**
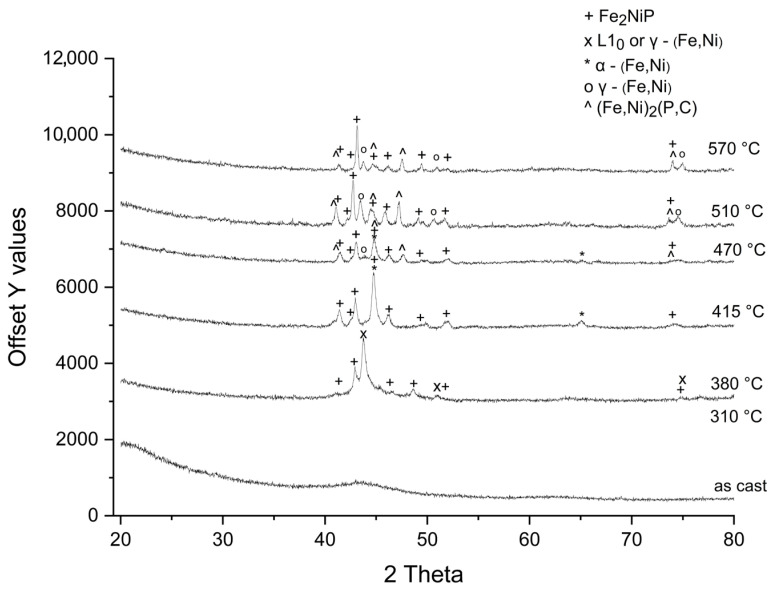
X-ray diffraction patterns of Fe_62_Ni_18_P_13_C_7_ ribbons annealed at given temperatures for 10 min in argon atmosphere, Cu-Kα radiation (λ = 1.5406 Å).

The X-ray diffractograms of the Fe_62_Ni_18_P_13_C_7_ annealed ribbons are presented in Figure 1. 

The ribbons remain fully amorphous (no crystalline diffraction peaks) up to an annealing temperature of 310 °C. Upon annealing at 380 °C, new diffraction peaks appear, indicating the nucleation of (Fe,Ni) and the Fe_2_NiP phases within the amorphous matrix. Due to overlapping fundamental peaks, it was not possible from XRD alone to unambiguously distinguish whether this intermediate (Fe,Ni) phase has the L1_0_ or fcc crystal structure. In samples annealed at 415 °C, the intermediate (Fe,Ni) phase is no longer detected. Instead, new diffraction peaks corresponding to the α-(Fe,Ni) phase appear, while those related to the Fe_2_NiP phase become more pronounced. At 470 °C, the γ-(Fe,Ni) phase is observed, coexisting with the α-(Fe,Ni) phase. Additionally, alongside the Fe_2_NiP phase, the crystallization of another phosphide phase—(Fe,Ni)_2_(P,C) is detected. At higher annealing temperatures (510 °C, 570 °C), the γ-(Fe,Ni) phase coexists with phosphides.

**Figure 2:**
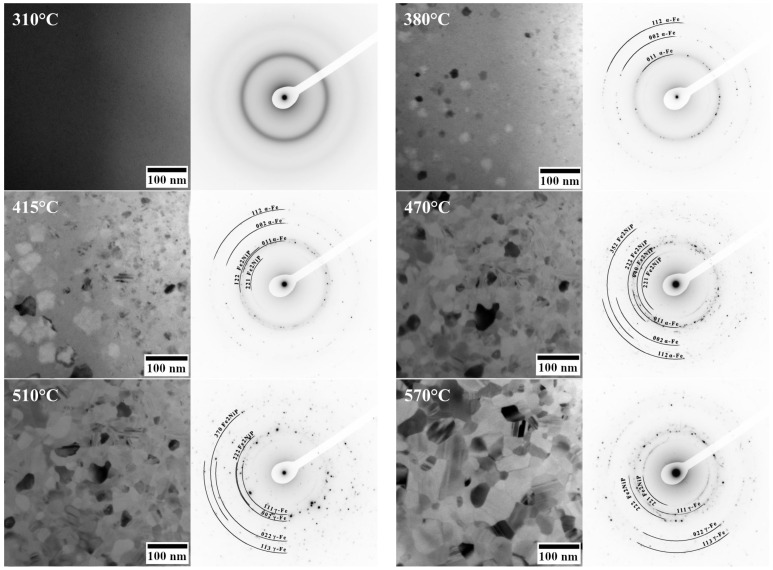
Selected area TEM images and corresponding Debye ring patterns recorded during in situ heating at a rate of 10 °C/min of Fe_62_Ni_18_P_13_C_7_ ribbons, illustrating the phase evolution from the amorphous state to crystalline phases at given temperatures.

In addition to conventional XRD measurements, in situ transmission electron microscopy (TEM) studies were carried out to monitor phase evolution during the devitrification process. The Debye ring patterns recorded during the heating are shown in Figure 2.

At 310 °C, only diffuse halos characteristic of the amorphous phase are observed (no crystalline spots or rings). Upon heating to 380 °C, the first crystallites nucleate within the amorphous matrix. The initial crystalline phase identified in the TEM at ~380 °C is α-(Fe,Ni). At 415 °C, the Fe_2_NiP phase appears and coexists with both the α-(Fe,Ni) solid solution and residual amorphous regions (regions between crystallites that have not yet interconnected). Further growth of the Fe_2_NiP phase is observed at 470 °C. At 510 °C, the α-(Fe,Ni) phase undergoes transformation into the γ-(Fe,Ni) phase. Twin boundaries, characteristic of austenitic phases, are clearly visible at this stage. At 570 °C, a significant increase in grain size is observed, although no additional new phases are detected. It is noteworthy that there is a discrepancy between the in situ TEM observations and the ex situ XRD results at the 380 °C stage: while XRD indicated the presence of an ordered (Fe,Ni) phase of L1_0_ or fcc structure, the in situ TEM (with a much shorter effective exposure at 380 °C) showed only α-(Fe,Ni) crystals.

**Figure 3:**
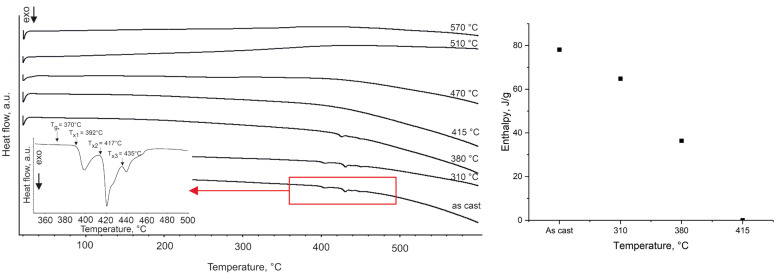
DSC curve of the Fe_62_Ni_18_P_13_C_7_ alloy, indicating the glass transition (*T_g_*), the onset of primary crystallization (*T_x1_*), the second (*T_x2_*) and third (*T_x3_*) crystallization events, along with the curves for ribbons annealed at the specified temperatures. Measurements were carried out at a heating rate of 10 °C/min in a nitrogen atmosphere (**left**). Evolution of crystallization enthalpy with annealing temperature (**right**).

The differential scanning calorimetry (DSC) curve of the investigated alloy is presented in Figure 3.

The glass transition region is narrow, and the width of the supercooled liquid region *ΔT_x_* (defined as $\Delta T_{x}=T_{x}-T_{g}$, where *T_g_* is the glass transition temperature and *T_x_* is the onset temperature of crystallization) is approximately 22 °C. Three exothermic peaks are observed, with the primary crystallization onset at *T_x_*_1_ ≈ 392 °C, a second crystallization event at *T_x_*_2_ ≈ 417 °C, and a third, smaller exothermic event at *T_x_*_3_ ≈ 435 °C. These multiple exotherms indicate a multistage devitrification process. Analysis of the crystallization enthalpies reveals a progressive decrease in the amorphous phase fraction as annealing temperature increases (Figure 3). The crystallization exothermic enthalpy of a ribbon annealed at 310 °C corresponds to an estimated ~83% remaining amorphous fraction, which drops to ~47% after annealing at 380 °C. Ribbons annealed at or above 415 °C show essentially no residual amorphous phase by this DSC criterion, consistent with those samples being fully crystalline.

### 3.2. Hardness Evolution

**Figure 4:**
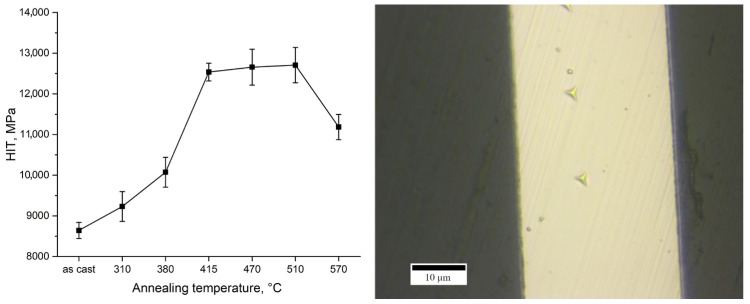
Indentation hardness (HIT) obtained from nanoindentation measurements of ribbons in the as-cast state and after annealing (10 min) (**left**) and an example of the obtained indents (**right**).

The evolution of hardness with annealing temperature is presented in Figure 4.

The hardness of the as-cast ribbon was approximately 8500 MPa. With increasing annealing temperature up to 415 °C, the hardness increases markedly, by about 50%, reaching roughly 12,500 MPa at 415 °C. In the intermediate temperature range between 415 °C and 510 °C, the hardness remains relatively constant, on the order of ~12,500–13,000 MPa. However, at the highest annealing temperature of 570 °C, a significant drop in hardness is observed, with the hardness decreasing by approximately 1500 MPa compared to the 510 °C condition. Thus, the hardness vs. temperature curve shows an initial rise, a plateau between ~415–510 °C, and then a decline at 570 °C.

### 3.3. SLM Printing

**Figure 5:**
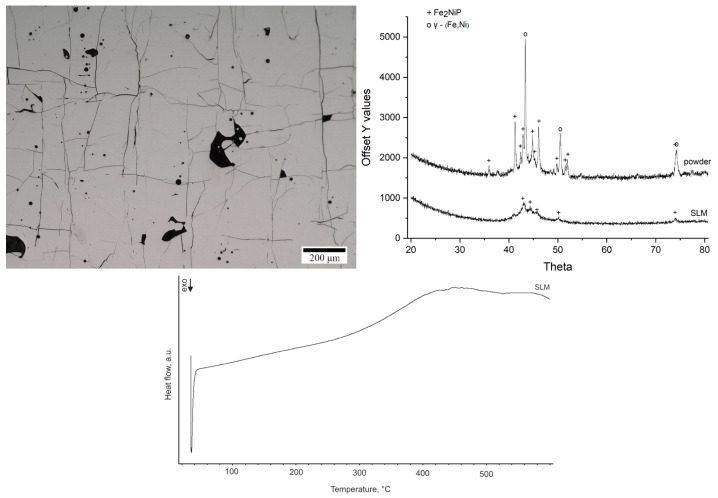
Microstructure of SLM printed sample (Keyence VHX-6000 digital microscope) (**left**); diffractogram of powder and printed sample, Cu-Kα radiation (λ = 1.5406 Å) (**right**); DSC curve of the printed sample at a heating rate of 10 °C/min in a nitrogen atmosphere (**bottom**).

The microstructure of the printed sample produced from Fe_62_Ni_18_P_13_C_7_ powder is shown in Figure 5.

It reveals a consistent, irregular square grid of cracks throughout the structure, accompanied by the presence of pores. The phase constitution of the feedstock powder and the SLM-fabricated sample was examined by XRD (Figure 5). The XRD pattern of the as-received powder shows that the alloy powder was predominantly crystalline prior to printing, with strong diffraction peaks corresponding to the γ-(Fe,Ni) and Fe_2_NiP phases. This observation is consistent with the low glass-forming ability of Fe_62_Ni_18_P_13_C_7_ alloy. After SLM processing, the XRD pattern of the printed sample still shows the presence of the Fe_2_NiP phase, although its diffraction peaks are less intense compared to the powder. Peaks corresponding to the γ-(Fe,Ni) phase are absent, suggesting that during laser melting and rapid solidification, part of the γ-phase present in the powder either partially dissolved or transformed, and Fe_2_NiP crystallized from the melt in its place. The DSC curve of the printed sample (Figure 5) indicates that the SLM sample is nearly fully crystallized. Only a very small residual exothermic signal is observed upon heating, which corresponds to an estimated ~1.9% amorphous fraction remaining in the printed material (calculated by comparing the crystallization enthalpy of the printed sample to that of a fully amorphous ribbon).

The measured hardness for the printed sample was 10,200 ± 600 MPa. This hardness value is consistent with a mostly crystalline material that still contains a minor fraction of amorphous phase or is very fine. The presence of a crack grid prevents further mechanical testing of the sample.

**Figure 6:**
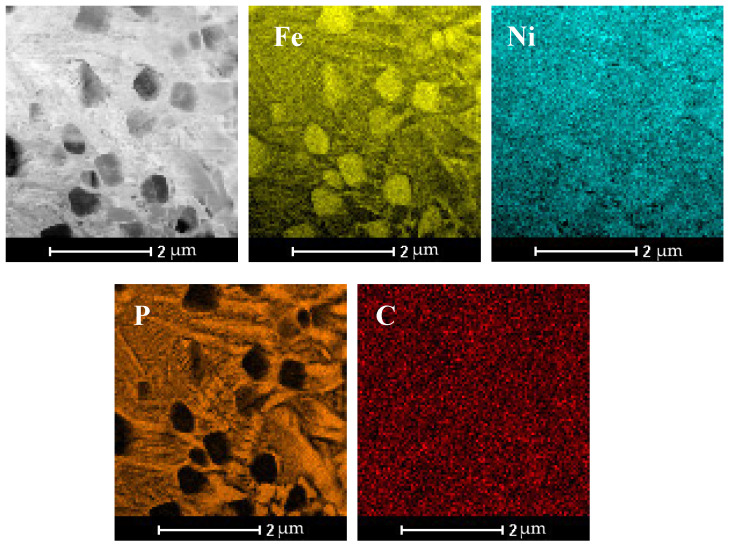
STEM microstructure and elemental composition maps in the Fe_62_Ni_18_P_13_C_7_ SLM printed sample.

**Figure 7:**
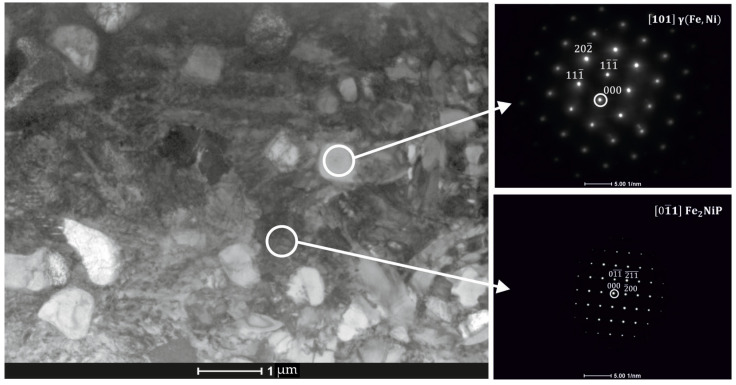
Transmission electron microscopy (TEM) image of Fe_62_Ni_18_P_13_C_7_ SLM-printed sample along with corresponding diffraction patterns identifying the γ-(Fe,Ni) solid solution and Fe_2_NiP phosphides.

Further microscopic analysis of the SLM sample was conducted using STEM-EDS mapping. Elemental mapping in Figure 6 reveals two distinct regions: (i) localized phosphorus-enriched regions with widths up to ~500 nm, although the majority were significantly narrower and (ii) iron-enriched regions with widths up to 600 nm. The phosphorus-enriched zones correspond to the Fe_2_NiP phase, and Fe-rich areas might be assigned to the γ-(Fe,Ni) type solid solution (Figure 7).

From the maps, it is evident that the volume fraction of the Fe_2_NiP phase is larger than that of the γ-(Fe,Ni) phase in these local areas, consistent with the pronounced P segregation. Overall, the microstructure of the SLM-fabricated alloy consists of a brittle Fe_2_NiP intermetallic network intermixed with regions of γ-(Fe,Ni) solid solution. Notably, α-(Fe,Ni) was not observed in the as-printed sample; the iron-rich phase present after SLM is in the γ-(Fe,Ni) form.

## 4. Discussion

### 4.1. Phase Transformation

Upon annealing the Fe_62_Ni_18_P_13_C_7_ ribbons, the alloy undergoes a multistage crystallization process. The XRD and TEM results together indicate that the first crystalline phase to form is around 380 °C. XRD suggests this phase could be the L1_0_-FeNi (tetrataenite) or fcc-FeNi (taenite), phase but distinguishing between these ordered structures by conventional XRD is challenging due to overlapping fundamental peaks and the requirement to observe superlattice reflections, which are typically very weak and often fall below the detection limit of conventional XRD measurements [23]. Tetrataenite (L1_0_-FeNi), if formed, is noteworthy because it is a highly ordered FeNi phase known for its exceptional magnetic properties [24]. It was first discovered in meteorites [25,26]. The L1_0_-crystal structure belongs to the P4/mmm space group and typically forms near equiatomic compositions (50:50 atomic%) in certain alloy systems. In most cases, the L1_0_-phase forms via a nucleation-and-growth mechanism from the disordered parent A1 (fcc) phase, below a critical chemical order-disorder temperature [27]. In Fe-Ni alloys, tetrataenite is typically obtained during slow cooling below ~320 °C, as atomic rearrangement from the disordered face-centered cubic taenite phase into a tetragonal distorted face-centered cubic cell requires long-range diffusion [28]. Under natural conditions, such as those found in meteorites, this process can take over 10^4^ years due to the extremely low diffusion rates at these temperatures [28]. In laboratory studies, alternative synthesis routes for tetrataenite have been explored, such as the annealing of metallic glasses [29] or cyclic oxidation-reduction of nickel-coated iron particles [30]. In the case of Fe-Ni-P-C alloys, the crystallization of tetrataenite is believed to be closely related to the presence of phosphorus. Phosphorus significantly enhances the mobility of nickel atoms, particularly at low temperatures in the range of 300–700 °C [31]. The model proposed by Ke et al. [31] predicts a tenfold acceleration of vacancy-mediated solvent diffusion at temperatures below 600 °C when the phosphorus content exceeds 0.1 at.%. Therefore, conditions are favorable for the formation of ordered Fe–Ni phases during annealing of this alloy, despite the kinetic constraints that usually prevent their formation on laboratory timescales.

The appearance of the intermediate (Fe,Ni) phase around 380 °C in the ribbons is accompanied by the onset of nucleation of the Fe_2_NiP intermetallic compound, so the (Fe,Ni)_3_P type phase. Fe_2_NiP (schreibersite) is a brittle intermetallic compound, with a reported hardness in the range of 800–950 HV [32]. Its formation contributes significantly to the embrittlement of metallic glasses upon crystallization.

By 415 °C, the XRD data show that the initial (Fe,Ni) phase present at 380 °C has disappeared, replaced by a mixture of α-(Fe,Ni) and phosphide phases. This suggests that the intermediate phase was metastable and decomposed upon further heating. At this stage, the predominant phases are α-(Fe,Ni), a ferromagnetic bcc phase, and Fe_2_NiP. At higher temperatures, the α-(Fe,Ni) phase partially transforms into the γ-(Fe,Ni) phase. The observed sequence of phase evolutions is in agreement with reported phase transformations in Fe–Ni–P alloys [33]. The stabilization of γ-(Fe,Ni) (fcc) over α-(Fe,Ni) (bcc) at elevated temperature is consistent with the Fe–Ni binary phase behavior, where higher Ni content and higher temperatures favor the fcc phase [34]. The crystallization pattern is relevant to the structural relaxation and embrittlement behavior of metallic glasses upon annealing. In particular, (Fe_100−x_Ni_x_)_83_B_17_ metallic glasses have been shown to exhibit embrittlement only in compositions that crystallize predominantly into the bcc phase [35]. In this study, the Fe_62_Ni_18_P_13_C_7_ ribbons crystallized primarily into α-(Fe,Ni) at lower annealing temperatures, which would suggest a risk of embrittlement. At higher temperatures, α-(Fe,Ni) transformed to γ-(Fe,Ni). The eventual presence of γ-phase (especially in combination with a fine microstructure) could mitigate some embrittlement, but in practice, the extensive precipitation of Fe_2_NiP likely dominates and causes brittleness regardless of α or γ matrix phase.

It is worth noting a discrepancy between the in situ TEM observations and the XRD results for samples annealed at 380 °C. While XRD suggests the formation of an L1_0_-FeNi or fcc-FeNi type phase, the in situ TEM analysis reveals only the presence of α-(Fe,Ni). This difference is likely attributable to the significantly shorter exposure times during the in situ TEM heating experiments and hence the lack of time for atoms to rearrange.

Thermal parameters from DSC reinforce the conclusion that Fe_62_Ni_18_P_13_C_7_ has a low glass-forming ability and multistage crystallization pattern. The glass-forming ability (GFA) of an alloy is often characterized parameters such as the critical cooling rate (R_c_) and critical casting diameter (D_c_), where R_c_ represents the minimum cooling rate required to suppress crystallization and achieve a fully amorphous structure, while D_c_ denotes the maximum sample dimension that allows for the formation of a completely amorphous phase [36]. Additionally, GFA can be estimated using various thermal parameters derived from DSC analysis [37]. One of the most commonly used indicators is the width of the supercooled liquid region (*ΔT_x_*) [37]. Alloys with a small, supercooled liquid region (*ΔT_x_*) require high cooling rates to form an amorphous structure and tend to crystallize easily upon any thermal input. The measured *ΔT_x_* of 22 °C matches well with literature data. For example, Ma et al. [16] reported the *ΔT_x_* of 23 °C for a 2.3 mm diameter rod cast from a similar alloy composition, Fe_80_Ni_20_P_13_C_7_. The fact that the atomized powder was largely crystalline (as shown by XRD) further illustrates the difficulty of retaining an amorphous structure in this composition without extremely high cooling rates or special processing. The DSC curve reveals multiple closely spaced exothermic events, which are indicative of a complex, multistage crystallization process. The first crystallization peak is most likely associated with the formation of the α-(Fe,Ni) phase, followed by a second event corresponding to the nucleation of the Fe_2_NiP intermetallic compound. The third exothermic peak is also observed at a higher temperature. Since no new phases are detected by TEM at 470 °C (Figure 2), this final thermal event is likely related to further ordering or the continued crystallization of the Fe_2_NiP phase that initially formed around *T_x_*_2_.

### 4.2. Hardness Evolution

Annealing has a pronounced influence on the hardness evolution of the Fe_62_Ni_18_P_13_C_7_ alloy. A ~50% increase in hardness between the as-cast state and samples annealed at 415 °C may be attributed to structural transformations occurring within this temperature range. Specifically, up to 415 °C, nanocrystallization takes place, leading to the formation of α-(Fe,Ni) nanocrystals, as well as the initial precipitation of Fe_2_NiP intermetallic, as supported by both XRD and TEM data. The development of these nanometer-scale phases induces classic dispersion strengthening: the presence of hard particles (1000 HV 0.05 for Fe_3_P and 1100 HV 0.05 for Fe_2_P [38]) and refined α-(Fe,Ni) grains effectively impedes plastic deformation, thereby elevating the overall hardness. This phenomenon aligns with the strengthening behavior observed in other partially crystallized metallic glasses [39,40,41]. Crystallite size grows from ~20 nm to ~50 nm between 380 °C and 415 °C (Figure 2), enhancing hardness via dispersion strengthening up to an optimal size (~50 nm) [40,41]. Between 415 °C and 510 °C, the alloy retains its elevated hardness, despite continued microstructural evolution. Between 415 °C and 470 °C, the amorphous fraction diminishes further, and the volume fraction of both α-(Fe,Ni) and Fe_2_NiP increases. At 470 °C, the alloy becomes fully crystalline, primarily consisting of α-(Fe,Ni) and Fe_2_NiP. Then, the α-(Fe,Ni) transforms to γ-(Fe,Ni). The α-Fe phase typically exhibits higher hardness than γ-Fe, and its hardness remains more stable with temperature [42]. Two opposing effects appear to balance each other: (i) the transformation of the harder α-phase to the softer γ-(Fe,Ni) phase begins around 470–510 °C, while (ii) the volume fraction of hard Fe_2_NiP continues to grow. The retention of significant α-phase content and the presence of additional phosphide precipitates likely offset the inherent softness of the emerging γ-phase, maintaining overall high hardness. At 570 °C, however, a marked drop in hardness is observed due to further reduction of α-phase content and grain coarsening.

### 4.3. SLM Printing

The SLM processing results provide insight into the challenges of additively manufacturing alloys with low GFA, such as Fe_62_Ni_18_P_13_C_7_. The printed samples suffered from extensive cracking, which is consistent with reports in the literature that Fe-based metallic glass forming alloys tend to crack during laser powder bed fusion [6]. The use of a random hatch remelting strategy was motivated by its previously reported effectiveness in reducing crystallinity in another low-GFA alloy, Fe_71_Si_10_B_11_C_6_Cr_2_ (at.%), as demonstrated by Zrodowski et al. [7]. In the present work, the two-stage scanning approach (premelt followed by remelt) similarly failed to produce a fully amorphous structure in the Fe_62_Ni_18_P_13_C_7_ alloy. Nonetheless, it appears to have influenced the resulting phase distribution: the printed sample retained a minor amorphous fraction (~1.9%) and exhibited a reduced fraction of the γ-(Fe,Ni) phase compared to the initial powder. Essentially, the laser parameters were sufficient to melt the powder and homogenize it briefly in the melt pool, but as the melt pool cooled and subsequently experienced reheating from nearby scan tracks, the alloy could not avoid crystallization.

The formation of a crack network in the SLM sample can be attributed to several factors. First, the alloy’s poor glass-forming ability means it crystallizes during solidification into brittle phases. Second, the rapid cooling and repeated thermal cycling in SLM (as new layers are deposited) generate high internal stresses. The combination of a hard, brittle microstructure and high thermal stresses is conducive to crack initiation and propagation.

The pores observed in the microstructure can be classified into two types according to [43]: (A) small, round pores, typically referred to as hydrogen or metallurgical porosity, and (C) rectangular or triangular pores with sharp edges. The small gas porosity (A) might be minimized through drying of the powder and maintaining a high-purity inert atmosphere. The second type of pores (C) is inherently linked to the thermal stresses exceeding the material’s strength, leading to fracture and material detachment during metallographic preparation. This is consistent with the observed high density of cracks observed in the printed sample.

One intriguing aspect of the SLM sample is the presence of the γ-(Fe,Ni) phase, as opposed to the α-(Fe,Ni) that was the primary phase in annealed ribbons. The difference arises from the processing paths: the gas-atomized powder was largely γ to begin with (due to its solidification condition), and the SLM process, despite partial remelting, did not convert a γ-(Fe,Ni) to α-(Fe,Ni). Some γ-(Fe,Ni) phase was retained or re-formed upon solidification of the melt pools. It has been reported in the literature that if the triggering substrate exhibits any crystallographic matching with the phase to be nucleated, it can influence primary phase selection [34], which might also explain the formation of the γ-(Fe,Ni) phase. An additional factor is the nickel content, at lower Ni contents, the (Fe,Ni) solid solution adopts a body-centered cubic (bcc) structure, whereas at higher Ni contents, it transforms into a cubic close-packed (ccp, γ-phase) structure in (Fe-Ni)-based metallic glasses [34]. Since the formation of Fe_2_NiP consumes more Fe, it may promote the formation of the γ-(Fe,Ni) phase. The Fe-Ni-P-C systems are susceptible to liquid spinodal decomposition, even during conventional casting [44], which favors the formation of phosphides. Sarac et al. [14] showed that introducing nanometer-sized γ-(Fe,Ni) precipitates in an Fe–Ni–P–C bulk glass could dramatically increase compressive plasticity, by effectively lowering the shear modulus of the matrix and promoting the formation of numerous shear bands.

In our SLM sample, we have a γ-phase present along with the phosphides, but this did not translate into any observable improvement in cracking resistance. The crucial difference is the length scale and distribution of phases: in Sarac’s work [14], the γ precipitates were very fine and uniformly dispersed in an otherwise amorphous matrix, which optimally tuned the mechanical response. In the SLM-processed material, the microstructure is coarse and the brittle phase Fe_2_NiP forms a continuous network at the grain boundaries of the γ-(Fe,Ni). Such a microstructure is extremely prone to cracking because the brittle phase provides easy crack propagation paths, and the γ-phase islands are too large to arrest or deflect cracks effectively.

Overall, the SLM experiment on Fe_62_Ni_18_P_13_C_7_ highlights the challenge of processing low-GFA alloys additively. The random remelting strategy, while somewhat effective in reducing crystallinity (as also observed in [7]), was insufficient to achieve an amorphous structure or suppress cracking. The resulting microstructure, characterized by decreased γ-phase content, increased Fe_2_NiP, and a minor amorphous fraction, remained predominantly crystalline and brittle. A comparative approach, based on the analysis of annealed ribbons and SLM-processed samples, shows that while controlled annealing enables the formation of a fine nanostructured composite with high hardness, SLM solidification leads to a coarse, phase-separated structure with inferior mechanical resilience. Bridging this microstructural gap is essential for the successful additive manufacturing of such alloys.

## 5. Conclusions

Based on the conducted research, the following conclusions can be drawn:

− The Fe_62_Ni_18_P_13_C alloy exhibits a narrow supercooled liquid region (*ΔT_x_* ≈ 22 °C) and readily crystallizes upon heating, indicating low glass-forming ability.
− The annealing process induces multistage crystallization in the alloy. At approximately 380 °C, α-(Fe,Ni) (or a metastable (Fe,Ni) phase that subsequently decomposes into α-(Fe,Ni) and Fe_2_NiP) and Fe_2_NiP phases are formed. With increasing temperature, α-(Fe,Ni) transforms into γ-(Fe,Ni), accompanied by further phosphide growth.
− Nanocrystallization increases hardness by ~50%, reaching ~12.5 GPa at 415 °C, due to the formation of fine α-(Fe,Ni) grains and Fe_2_NiP precipitates. Grain coarsening above 510 °C reduces hardness.
− SLM processing results in a predominantly crystalline, brittle microstructure with only ~2% amorphous content and visible thermal cracking. Hardness of the as-printed sample (~10.2 GPa) reflects its phase composition.

## Data Availability

The original contributions presented in the study are included in the article, further inquiries can be directed to the corresponding author.
